# Supplementary material for: The Systems Biology Research Tool: evolvable open-source software
Source: BMC Syst Biol. 2008 Jun 29;2:55. doi: 10.1186/1752-0509-2-55 (PMC2446383; doi:10.1186/1752-0509-2-55)
Supplement: Additional file 1 — SBRT Archive. An archive of the current version of the Systems Biology Research Tool. [file 1752-0509-2-55-S1.zip › sbrt-1.4.0/doc/developers_guide/api/sbrt/shell/mng/algebra/package-summary.html]

sbrt.shell.mng.algebra


|  |  |  |
| --- | --- | --- |
| **Overview** \| **Package** \| Class \| **Use** \| **Tree** \| **Deprecated** \| **Index** \| **Help** | |  |
| **PREV PACKAGE**   **NEXT PACKAGE** | **FRAMES**    **NO FRAMES**     **All Classes** |  |


---

## Package sbrt.shell.mng.algebra

Provides classes for creating algebra process managers.

**See:**
  
          **Description**

| **Class Summary** | |
| --- | --- |
| **LinearCombConverterManager** | This class is used to convert linear combinations to matrices. |
| **LinearSolverManager** | This class is used to manage linear system solvers. |
| **MatrixConverterManager** | This class is used to convert matrices to linear combinations. |
| **MultipleVectorsConverterManager** | This class is used to convert files containing multiple vectors into a series of files, each containing a single vector. |
| **SingleVectorConverterManager** | This class is used to convert multiple files containing single vectors into a single file containing multiple vectors. |

## Package sbrt.shell.mng.algebra Description

Provides classes for creating algebra process managers.

---


|  |  |  |
| --- | --- | --- |
| **Overview** \| **Package** \| Class \| **Use** \| **Tree** \| **Deprecated** \| **Index** \| **Help** | |  |
| **PREV PACKAGE**   **NEXT PACKAGE** | **FRAMES**    **NO FRAMES**     **All Classes** |  |


---
